# Clinical outcomes of adults accessing acute medical same-day emergency care in the NHS: a retrospective cohort study across two hospitals

**DOI:** 10.1136/bmjopen-2026-121412

**Published:** 2026-07-01

**Authors:** Sue Dean, Jo Leonardi-Bee, Julian Barratt, Holly Blake

**Affiliations:** 1 Same Day Emergency Care, United Lincolnshire Teaching Hospitals NHS Trust, Boston, UK; 2 School of Health Sciences, University of Nottingham Faculty of Medicine and Health Sciences, Nottingham, UK; 3 Centre for Evidence Based Healthcare, University of Nottingham, Nottingham, UK; 4 Centre for Advancing Practice, Aston University, Birmingham, UK; 5 Aston Medical School, Aston University, Birmingham, UK; 6 University of Wolverhampton, Wolverhampton, UK; 7 Faculty of Medicine and Health Sciences, University of Nottingham, Nottingham, UK

**Keywords:** ACCIDENT & EMERGENCY MEDICINE, Delivery of Health Care, Integrated, Emergency Service, Hospital, HEALTH SERVICES ADMINISTRATION & MANAGEMENT, INTERNAL MEDICINE

## Abstract

**Abstract:**

**Objectives:**

Same-day emergency care (SDEC) has been rolled out as a model of care in England with a limited evidence base. This study examined conversion to inpatient admission, 30-day reattendance and 30-day mortality, among adults managed through SDEC compared with those admitted for ≤48 hours, as a proxy for low acuity, over a 4-year period, to assess safety and effectiveness in a real-world operational setting across two acute hospital sites.

**Design:**

Retrospective cohort study.

**Setting:**

Two acute hospital sites within one National Health Service (NHS) trust in England, UK.

**Participants:**

Adults aged ≥18 years attending acute medical services between April 2021 and March 2025, managed via SDEC or admitted for ≤48 hours (n=43 970).

**Outcome measures:**

Conversion to inpatient admission, 30-day reattendance and 30-day mortality.

**Results:**

The crude conversion rate from SDEC to inpatient admission was 5.8%. In the multivariable model, increasing age (OR 1.02, 95% CI 1.01 to 1.02), male sex (OR 1.42, 95% CI 1.29 to 1.57) and attendance at the Boston site (OR 1.71, 95% CI 1.55 to 1.90) were associated with higher odds of admission.

30-day prefix-concordant reattendance occurred in around 10% of patients in both pathways. After adjustment, SDEC patients had substantially lower odds of reattendance than those admitted for ≤48 hours (OR 0.26, 95% CI 0.19 to 0.36). The effect of SDEC varied by age (interaction OR 1.02, 95% CI 1.01 to 1.02) and site, with a weaker protective effect at Lincoln compared with Boston (interaction OR 2.12, 95% CI 1.74 to 2.60). Age was associated with a reduction in reattendance (OR 0.99 per year increase, 95% CI 0.98 to 0.99).

30-day mortality was lower in SDEC than in short-stay admission (0.6% vs 8.2%), with pathway remaining a strong predictor after adjustment (OR 0.05, 95% CI 0.04 to 0.07). Younger age was protective, while male sex was associated with higher mortality. Pathway by sex interaction indicated a less pronounced protective effect of SDEC in men.

**Conclusions:**

SDEC was associated with very low conversion to inpatient admission and substantially better short-term outcomes than short-stay admission, including markedly reduced diagnosis-concordant reattendance and lower 30-day mortality. These findings indicate that SDEC is a safe and effective model for managing selected acute medical patients. The consistently favourable outcomes among SDEC attenders suggest that patient selection and operational factors within the emergency pathway may be directing lower-risk patients towards SDEC, highlighting the need to review how SDEC capacity is targeted to ensure alignment with its intended clinical role.

**STRENGTHS AND LIMITATIONS OF THIS STUDY**

- Consistently applied inclusion criteria and rigorous data management procedures strengthened internal validity and supported reliable findings from multivariable modelling.
- Sensitivity analyses indicated robustness of findings and assessed the impact of key methodological assumptions.
- Use of routinely collected administrative data introduced limitations related to coding accuracy, restricted variable availability and the potential for unmeasured confounding.
- Case identification was incomplete, as acute medical patients managed entirely within the emergency department were not coded to the acute medical admission pathway, leading to under-representation of short-stay admissions.
- Outcome measurement may have been limited by incomplete capture of mortality and reattendance events occurring outside the trust, although this is likely minimal given the county’s geography, and by the absence of clinical observations at presentation, requiring short-stay admission to be used as a proxy for patient acuity.

## Introduction

 Same-day emergency care (SDEC) is now a core component of acute medical services across the NHS, formalised through national policy from 2019 onwards,^1^ and endorsed by invested professional bodies.^2 3^ SDEC provides rapid assessment, diagnostics and treatment for patients who might otherwise require admission, improves flow and reduces pressure on emergency departments (EDs) and inpatient beds. Despite national guidance, substantial variation persists in how SDEC is organised, the conditions it manages, and the criteria used to select patients.^4^,^6^ Internationally, comparable models exist, such as ambulatory emergency care units in Europe and observation medicine units in the USA, offering alternative approaches to managing lower-acuity medical presentations.

Evidence describing real-world SDEC activity and outcomes remains limited.^4^,^6^ Existing studies are often small, single-centre or condition-specific, and few compare outcomes between SDEC attendances and short-stay medical admissions.^7 8^ As a result, the extent to which SDEC achieves its intended aims, reducing admissions, preventing reattendance and supporting safe same-day discharge, remains uncertain.

SDEC operates within a wider urgent and emergency care (UEC) system characterised by sustained ED crowding and bed scarcity.^9 10^ National SDEC specifications now recommend that 20%–30% of SDEC attendances should result in admission.^11^ However, operational pressures may incentivise the diversion of lower-acuity patients to SDEC, influencing case-mix and complicating interpretation of outcomes.

This study aimed to examine conversion to inpatient admission, 30-day reattendance and 30-day mortality among adults accessing acute medical SDEC across two hospital sites over a 4-year period.

## Methods

### Study design and setting

This retrospective observational twin-centre cohort study was conducted at United Lincolnshire Teaching Hospitals NHS Trust (ULTH), using data from the acute medical SDEC services at Lincoln and Boston hospitals. The study examined activity across both SDEC units and the corresponding acute medical inpatient pathways. SDEC was operational for 12–14 hours a day throughout the study period. Only the first SDEC attendance was included, with follow-up visits being excluded. Planned follow-up reviews arranged by SDEC clinicians were excluded at source, whereas unplanned reattendance within 30 days was captured as an outcome.

Lincoln County Hospital, in the urban centre of Lincoln, and Pilgrim Hospital Boston, serving a predominantly rural and coastal population, provide contrasting geographical and demographic contexts. Both hospitals act as county-wide hubs for selected specialist services and receive acute medical referrals from EDs, the ambulance service and primary care. Patients were directed to SDEC following initial triage in ED or via direct ambulance or general practitioner referral, according to local eligibility criteria and clinical judgement. Patients were accepted for SDEC if they were aged 18 years or over, ambulant or able to mobilise with minimal assistance, had capacity to participate in assessment and decision-making, a National Early Warning Score (NEWS2) score of ≤5, with no single parameter scoring 3 (ie, they were physiologically stable), and a presumed acute medical problem suitable for same-day investigation and treatment. Patients not meeting these criteria, including those with severe physiological derangement, need for continuous monitoring, or clear indications for inpatient admission, were referred directly to the acute medical team for admission.

Administrative hospital data derived from the Trust’s Patient Administration System, which is routinely submitted to Hospital Episode Statistics, were used to identify all acute medical SDEC attendances and all acute medical admissions during the study period. Diagnostic information was coded using the International Classification of Diseases, 10th Revision (ICD-10).^12^ Clinical severity measures such as NEWS2 and frailty scores (eg, Clinical Frailty Scale) are recorded in paper notes within the Trust; however, they are not captured in electronic systems and therefore could not be extracted at scale for this study. As a result, physiological acuity and frailty could not be included in the analysis.

### Data sources and variables

Administrative hospital data were obtained from ULTH’s admitted patient database for all acute medical SDEC attendances and admissions over 4 full years from 1 April 2021 to 31 March 2025. Extracted variables included demographics, hospital site, admission and discharge dates and times, ward codes, discharge destination and primary ICD-10 diagnosis codes. Linked hospital episode data identified 30-day reattendance and 30-day mortality. Repeat attendances after the index visit, and SDEC visits undertaken solely for postdischarge review, were excluded at source and therefore not present in the dataset.

Conversion to admission was defined as any inpatient admission following an initial SDEC assessment, and all such conversions were included when calculating the full conversion-to-admission rate, regardless of eventual length of stay. The number of SDEC conversions differs from the final pathway counts because conversions with a length of stay >48 hours were excluded from the short-stay analytic cohort, and final pathway grouping was based on ultimate disposition rather than initial pathway.

For analyses of 30-day reattendance and 30-day mortality, the cohort was restricted to patients with a length of stay ≤48 hours. Such short-stay admissions (≤48 hours) were used as a pragmatic proxy for lower acuity because clinical observations and frailty scores were not available in extractable electronic form. Within this short-stay analytic cohort, patients were grouped by final pathway, with patients who converted from SDEC to inpatient admission classified within the admitted group. Ethnicity was grouped into three categories (White British; Other ethnicity and unknown), and sex was analysed as recorded.

### Study population

The study included all acute medical patients across both sites, grouped into SDEC attendances and acute medical admissions with a length of stay ≤48 hours over a 4-year period to March 2025.

Adults aged 18 years and over were eligible for inclusion. All records contained complete identifiers and valid admission and discharge timestamps, and no cases were excluded due to missing data.

### Outcomes

Three primary outcomes were assessed to evaluate the safety and effectiveness of the SDEC pathway:

Conversion from SDEC to inpatient admission, defined as any SDEC episode resulting in transfer to an inpatient ward during the same attendance.

30-day reattendance for the same diagnosis, identified through linked hospital episode data and defined using ICD-10 prefix matching (a three-character code match between the index and subsequent diagnoses). Prefix-concordant matching was used to capture clinically related reattendance while allowing for variation in fourth-character coding.

30-day all-cause mortality, defined as death occurring within 30 days of discharge from the index episode. Mortality data captured deaths recorded within hospital; deaths occurring outside the Trust were not available in the administrative dataset.

### Statistical analysis

Data were cleaned and checked for internal consistency. Baseline demographic characteristics were described according to the patient’s initial management pathway (SDEC vs direct admission). Descriptive statistics were generated for the overall cohort and subgroups. Categorical variables were summarised as frequencies and percentages, and continuous variables as means (with SD) or medians (with IQR). Group comparisons used t-tests or Wilcoxon rank-sum tests for continuous variables and χ² or Fisher’s exact tests for categorical variables. Comparisons were made between initial pathways (SDEC vs ≤48-hour admitted), and hospital site was included as a covariate in all regression models. Because only two hospital sites were involved, potential intracluster correlation was addressed by specifying site as a fixed factor in modelling, because random-effects modelling would not be reliable with only two clusters. Statistical significance was defined as p<0.05. All analyses were conducted using R^13^ via RStudio.^14^ Given the nature of the study, no sample size calculation was required. Potential sources of bias, including pathway selection, incomplete capture of events occurring outside the trust, and limitations inherent to routinely collected data, were considered.

### Conversion to admission

Conversion to admission was defined as a transfer from SDEC to an inpatient ward within the same attendance. Monthly conversion rates were summarised descriptively, and logistic regression was used to examine factors associated with conversion. Univariate models estimated crude associations, followed by multivariable models including covariates selected a priori for clinical relevance and univariate significance. Age was retained as a continuous variable based on model fit, and a reduced model was derived by retaining predictors that remained significant in the full model.

### 30-day prefix-concordant reattendance

30-day reattendance was defined as any emergency hospital attendance or admission within 30 days of the index attendance. Only prefix-concordant reattendance (subsequent attendances with an ICD-10 code sharing the same three-character prefix as the index diagnosis) was analysed. Group comparisons used Wilcoxon rank-sum tests for continuous variables and Chi-squared tests for categorical variables. Logistic regression was used to model prefix-concordant reattendance, with multivariable models adjusting for sex, age (continuous), ethnicity and site. Interaction terms were assessed, and two (pathway×age and pathway×site) were retained as they materially influenced the pathway effect. The final model included pathway, age, sex, ethnicity, site and the retained interactions.

### 30-day all-cause mortality

30-day all-cause mortality was defined as death within 30 days of attendance. Group comparisons used Wilcoxon rank-sum tests for continuous variables and χ² or Fisher’s exact tests for categorical variables. Age was modelled categorically due to non-linearity. Logistic regression estimated crude and adjusted associations with mortality, with multivariable models adjusting for age group, sex, ethnicity and site. A reduced model was developed by excluding covariates that were neither associated with mortality nor predictors. Interaction terms were tested, and only the pathway×sex interaction was retained as it materially altered the pathway effect. The final model included pathway, age group, sex and the pathway×sex interaction.

### Patient and public involvement

Patients and members of the public were involved in the design and refinement of the study protocol. They were not involved in data collection, analysis or interpretation, but will be informed of the study findings through planned dissemination activities.

## Results

**Figure 1:**
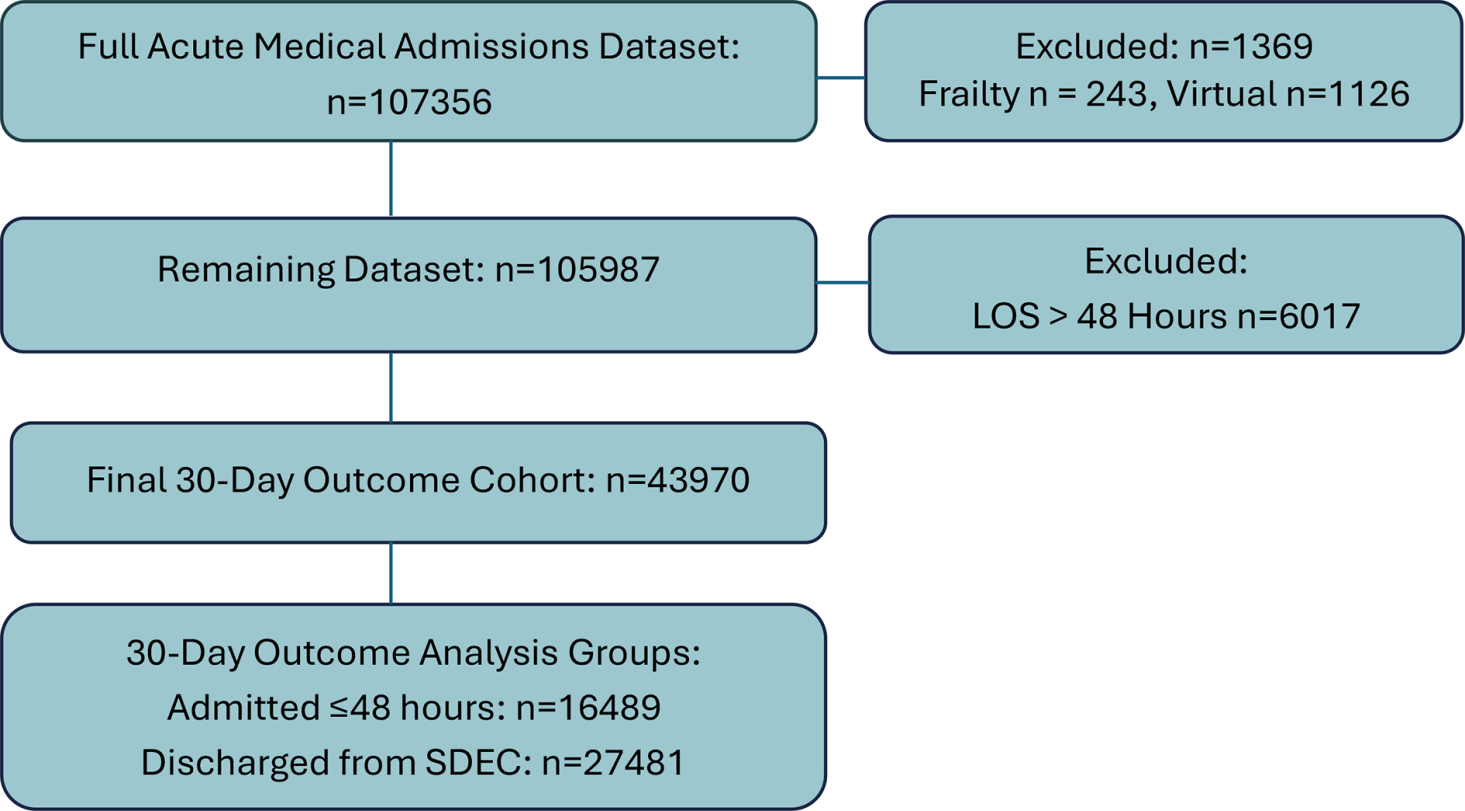
Flow chart of study population and the short-stay analytical cohort used for 30-day outcomes. LOS, length of stay; SDEC, same-day emergency care.

A total of 107 356 attendances were identified during the study period. After excluding 1369 frailty and virtual SDEC attendances and 62 017 attendances that did not meet the short-stay pathway criteria, 43 970 patients remained eligible for analyses and formed the short-stay analytic cohort (figure 1). This cohort comprised patients who were discharged the same-day from SDEC or admitted for ≤48 hours, including those admitted following an initial SDEC assessment. Of these, 27 481 were discharged directly from SDEC and 16 489 were admitted for ≤48 hours.

### Baseline characteristics

The short-stay analytic cohort included patients initially managed in SDEC and those admitted for ≤48 hours. Patients managed in SDEC were younger than those admitted.

Most patients were White British, and smaller proportions were recorded as unknown or other ethnicity. Patients from non-White or unknown ethnic backgrounds were more frequently managed via SDEC.

Sex distribution was balanced overall but females were more frequently managed in SDEC, whereas males were more evenly distributed across pathways.

Across the two hospital sites, SDEC use was higher at Lincoln than Boston. Full baseline characteristics are presented in table 1.

**Table 1:** Demographic characteristics and site by pathway

Variable	Category	Overall n (%)	SDEC n (%)	Admitted n (%)
Total	–	**43 970** (**100.0%**)	**27 835** (**63.3%**)	**16 135** (**36.7%**)
Age	Mean (SD)	58 (20.5)	55 (19.9)	63 (20.4)
Ethnicity	Other ethnicity	2561 (5.8)	1816 (70.9)	745 (29.1)
	Unknown	6547 (14.9)	4542 (69.4)	2005 (30.6)
	White British	34 862 (79.3)	21 477 (61.6)	13 385 (38.4)
Sex	Female	23 733 (54.0)	15 727 (66.3)	8006 (33.7)
	Unknown	70 (0.2)	37 (52.9)	33 (47.1)
	Male	20 167 (45.9)	12 071 (59.9)	8096 (40.1)
Site	Boston	20 367 (46.3)	12 192 (59.9)	8175 (40.1)
	Lincoln	23 603 (53.7)	15 643 (66.3)	7960 (33.7)

Where percentages do not equal 100% exactly, this is due to rounding.

SDEC, same-day emergency care.

The distribution of presenting diagnoses differed between pathways, with the most common conditions spanning respiratory, cardiac and infection-related categories (online supplemental figure 1).

### Outcomes

#### Conversion to admission

**Figure 2:**
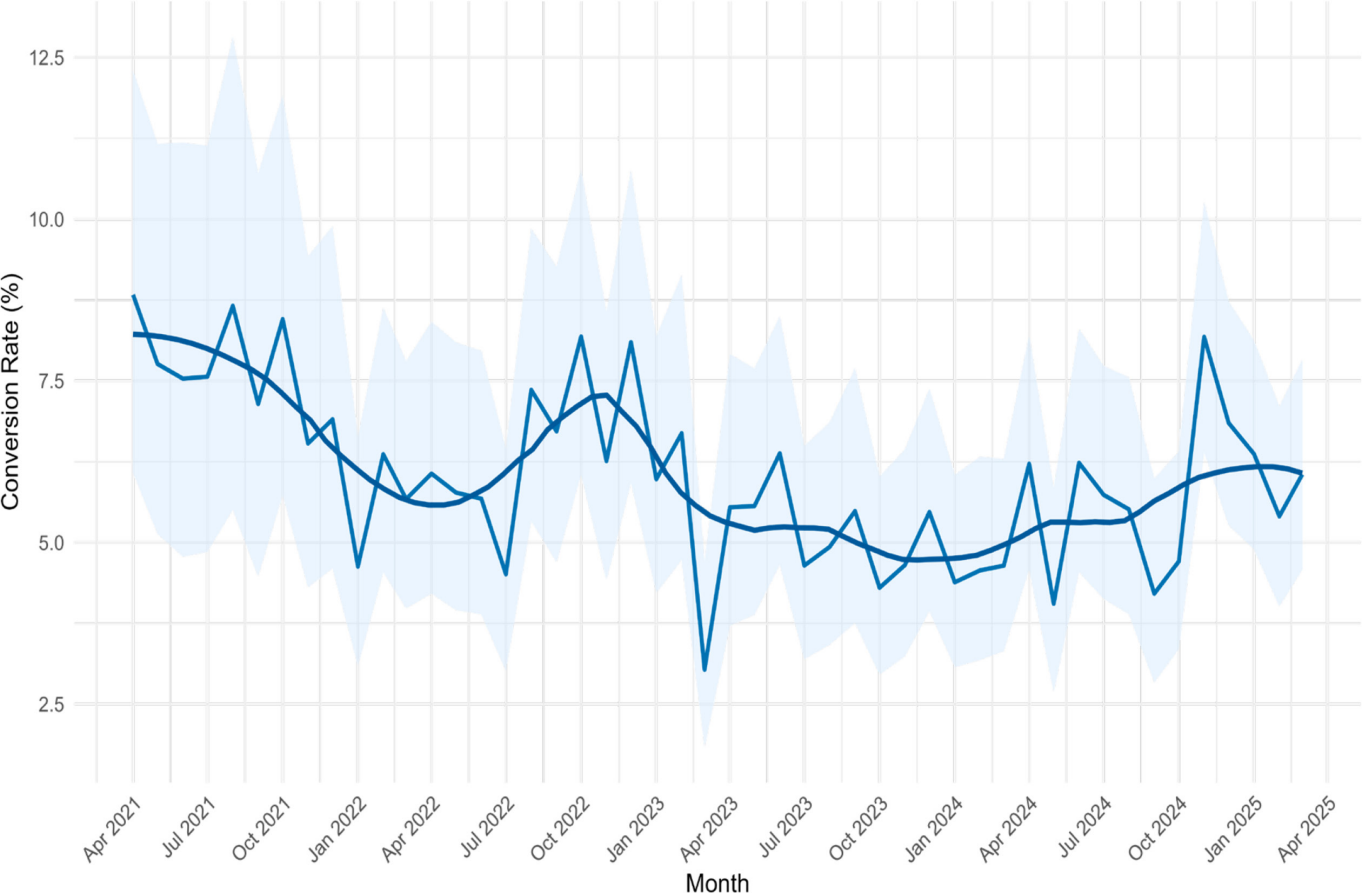
Conversion to admission over time.

Among patients initially managed through the SDEC pathway, conversion to inpatient admission was low overall. Conversion rates varied over time, with monthly fluctuations and an overall downward trend (figure 2). A smoothed trend suggested a gradual reduction in conversion likelihood from mid-2022 onwards.

In unadjusted analyses, male sex and increasing age were associated with higher conversion likelihood. In adjusted analyses, conversion was more likely among older patients, men and those attending the Boston site (table 2).

**Table 2:** Multivariable logistic regression for conversion to admission: final reduced model

Variable	Category	OR (95% CI)	P value
Sex	Male	1.42 (1.29 to 1.57)	**<0.001**
Age	Continuous	1.02 (1.01 to 1.02)	**<0.001**
Site	Boston	1.71 (1.55 to 1.90)	**<0.001**

The final model includes only predictors that were statistically significant in the full multivariable model (age, sex and site). Ethnicity was removed due to non-significance. ORs represent the adjusted association with 30-day reattendance. Reference categories: female (sex) and Lincoln (site). Robust SEs were used.

Bold indicates statistical significance at p<0.05.

#### 30-day prefix-concordant reattendance

Prefix-concordant 30-day reattendance rates were similar between pathways (9.9% SDEC vs 10.0% admitted).

Among patients who reattended with concordant diagnoses, reattenders originally admitted were older than those managed in SDEC. Sex distribution differed modestly, with females more frequently represented in the SDEC reattendance group.

Ethnicity distributions showed modest variation, with slightly higher proportions of other ethnicity and unknown groups in the SDEC cohort. Age group differences were marked, with a greater proportion of older reattenders in the admitted cohort.

Site differences were substantial: a higher proportion of admitted reattenders were from Boston, whereas SDEC reattenders were more often from Lincoln (table 3).

**Table 3:** Characteristics of readmitted patients by pathway and prefix-level diagnosis match

Variable	Category	SDEC	Admitted ≤48 hours	Test statistic	P value
Age	Median (IQR)	61 (42–75)	67 (49–79)	W=2 565 447	**<0.001**
	Mean (SD)	58.5 (19.9)	63.5 (19.7)		
Sex				χ²=8.7	**0.013**
	Female	1474 (53.5%)	795 (48.9%)		
	Male	1274 (46.3%)	827 (50.9%)		
	Other/ unknown	5 (0.2%)	3 (0.2%)		
Ethnicity				χ²=8.6	**0.014**
	Other ethnicity	149 (5.4%)	73 (4.5%)		
	Unknown	326 (11.8%)	153 (9.4%)		
	White British	2278 (82.7%)	1399 (86.1%)		
Site	Boston	932 (33.9%)	889 (54.7%)	χ²=182.1	**<0.001**
	Lincoln	1821 (66.1%)	736 (45.3%)		

Includes patients readmitted within 30 days following an index attendance or admission via SDEC or admitted ≤48 hours pathways. Diagnosis match is based on ICD-10 prefix concordance between index and reattendance episodes. Percentages are column-wise. Wilcoxon rank-sum test used for continuous age; χ2 tests used for categorical comparisons.

Bold indicates statistical significance at p<0.05.

ICD-10, International Classification of Diseases, 10th Revision; SDEC, same-day emergency care.

After adjustment, SDEC attendance was associated with lower adjusted odds of prefix-concordant reattendance, with the pathway effect varying by age and site (table 4).

**Table 4:** Multivariable logistic regression model for same-prefix reattendance within 30 days, including interaction effects

Variable	Category	OR (95% CI)	P value
Pathway	SDEC	0.26 (0.19 to 0.36)	**<0.001**
Age		0.99 (0.98 to 0.99)	**<0.001**
Site	Lincoln	0.97 (0.83 to 1.14)	0.744
Sex	Male	1.06 (0.96 to 1.16)	0.241
	Other/unknown	0.92 (0.23 to 2.50)	0.895
Ethnicity	Unknown	0.87 (0.69 to 1.11)	0.248
	White British	1.16 (0.95 to 1.44)	0.154
Pathway SDEC: age		1.02 (1.01 to 1.02)	**<0.001**
Pathway SDEC: Site Lincoln		2.12 (1.74 to 2.60)	**<0.001**

ORs and 95% CIs are derived from a multivariable logistic regression model assessing predictors of prefix-concordant reattendance among short-stay patients, including interaction terms. The reference categories were: female (sex), non-White/non-unknown (ethnicity), Boston site and ≤48-hour admission (pathway). Robust SEs were used.

Bold indicates statistical significance at p<0.05.

SDEC, same-day emergency care.

#### 30-day all-cause mortality

30-day all-cause mortality was markedly lower among patients managed in SDEC than among those admitted for ≤48 hours (0.6% vs 8.2%).

Among patients who died within 30 days, those originally admitted were older than those managed in SDEC. A higher proportion of males were among SDEC deaths. Ethnicity distributions were similar between pathways, while site differed, with a greater proportion of SDEC deaths occurring at Lincoln (table 5).

**Table 5:** Demographic and pathway characteristics of short-stay patients who died within 30 days of attendance

Variable	Category	SDEC	Admitted	Test statistic	P value
Age	Median (IQR)	75 (67–82)	80 (71–87)	W=143 131	**<0.001**
	Mean (SD)	73.3 (12.9)	77.7 (12.6)		
Age group	≤75	88 (50.9%)	492 (36.2%)	χ²=13.4	**<0.001**
	>75	85 (49.1%)	866 (63.8%)		
Sex				Fisher’s exact	**0.035**
	Female	63 (36.4%)	610 (44.9%)		
	Male	110 (63.6%)	748 (55.1%)		
	Indeterminate/unknown	0 (0%)	0 (0%)		
Ethnicity				Fisher’s exact	0.584
	White British	147 (85%)	1181 (87%)		
	Other Ethnicity	26 (15%)	174 (12.8%)		
	Unknown	0 (0%)	3 (0.2%)		
Site	Lincoln	108 (62.4%)	664 (48.9%)	Fisher’s exact	**<0.001**
	Boston	65 (37.6%)	694 (51.1%)		

Percentages are calculated within each pathway group (admitted vs SDEC) and reflect column-wise distributions. Age is summarised using both median and IQR as well as mean and SD. Statistical comparisons were performed using the Wilcoxon rank-sum test for continuous age and either χ2 or Fisher’s exact tests for categorical variables, depending on cell counts and expected frequencies. Categories with zero counts are retained for completeness.

Bold indicates statistical significance at p<0.05.

SDEC, same-day emergency care.

SDEC attendance was associated with substantially lower adjusted odds of 30-day mortality, with lower mortality in younger patients and higher mortality among men, and the pathway effect varied by sex (table 6).

**Table 6:** Multivariable logistic regression model for 30-day mortality, including the pathway×sex interaction

Variable	Category	OR (95% CI)	P value
Pathway	SDEC	0.05 (0.04 to 0.07)	**<0.001**
Age group	≤75	0.20 (0.15 to 0.26)	**<0.001**
Sex	Male	1.28 (1.14 to 1.43)	**<0.001**
Pathway SDEC: sex male		1.83 (1.32 to 2.56)	**0.001**

ORs are from the final multivariable logistic regression model, which included the pathway×sex interaction. Reference categories: admitted pathway, age group >75 years, sex female.

Bold indicates statistical significance at p<0.05.

SDEC, same-day emergency care.

## Discussion

This study examined conversion to admission, 30-day ICD-10 prefix-concordant reattendance and 30-day all-cause mortality to assess the safety and effectiveness of SDEC within a pressured UEC system, using short-stay acute medical admissions as a low-acuity comparison group.

Conversion to admission from SDEC was low overall, though varied by age, sex and site in crude analyses. Older adults and men had higher conversion rates descriptively, consistent with frailty, multimorbidity and gendered patterns of illness behaviour influencing clinical decision-making.^15^,^17^ In the adjusted model, age and sex remained associated with conversion, indicating that demographic factors contribute to variation in admission likelihood beyond case-mix differences. Site-level variation was the strongest influence, with substantially higher odds of admission at the Boston site, suggesting that organisational structures, operational thresholds, local workflow pressures and population characteristics may act as key mechanisms shaping pathway allocation. Importantly, both hospitals operated under the same Standard Operating Procedure (SOP) for SDEC, indicating that these differences arose not from formal criteria but from how the SOP was enacted under differing operational pressures, ED streaming behaviour and workflow routines. This highlights that pathway performance is shaped by local context even when policy is standardised.

A further contextual factor is the policy tension between ED pressures^9 10^ and national SDEC service specifications.^1 11^ EDs operate under intense pressure to reduce crowding, creating strong incentives to refer lower-acuity patients to SDEC to decompress the department, including many who do not require acute medical specialty input. These ED-streamed patients are typically low-acuity and are discharged the same day, which increases the overall SDEC discharge rate but broadens the case-mix beyond the intended scope of a criteria-based ambulatory model. In contrast, national guidance recommends that SDEC should admit 20%–30% of patients.^11^ This creates a practical tension: ED incentives drive conversion rates down, while SDEC policy frameworks anticipate higher admission proportions. The resulting divergence reflects competing organisational priorities rather than pathway performance alone, illustrating how system-level pressures shape pathway mechanisms. Conversion rates also varied over time, reflecting dynamic operational pressures and shifting referral patterns across the study period. NHS England^18^,^20^ have recently launched new guidance intended to clarify the roles of ED and SDEC within UEC. While this paragraph describes national-level pressures, the marked differences between the two study hospitals indicate that local operational practices also strongly influence how national policy is enacted.

Patient selection for SDEC is not standardised across the NHS, and services adopt different approaches. Some operate an inclusive, process-driven model in which all patients meeting broad SDEC criteria are accepted.^4 7^ Others use condition-specific pathways or front-door streaming, where ED triage staff decide who should be directed to SDEC and ‘inform and send’.^21^ Risk-stratification tools have also been trialled to support referral decisions, but these have shown limited sensitivity and specificity,^22 23^ and a multivariable prediction model remains in development.^24^ These contextual influences impact on patient selection for SDEC and are driven by a wider lack of consensus around structured patient selection versus clinical judgement. In the present study, patient selection differed in practice due to variation in ED flow pressures and local referral behaviour. This indicates that even when criteria are standardised, operational context determines who enters the pathway and how it performs.

30-day prefix-concordant reattendance occurred in around one in ten patients, with similar crude rates across pathways. In the adjusted interaction model, SDEC attendance was associated with lower odds of clinically related reattendance than short-stay admission, supporting the safety of same-day discharge. The SDEC effect varied by age, with differences between pathways narrowing among older adults, and by site, with higher prefix-concordant reattendance among SDEC patients at Lincoln than at Boston, indicating that local organisational context shapes pathway performance. These interactions indicate that the effect of pathway allocation is not uniform across patient groups, reinforcing the importance of contextual and organisational factors. Increasing age showed a small inverse association with reattendance, while sex and ethnicity were not independently associated. The site differences observed are consistent with variation in how the SOP was operationalised locally, particularly in relation to ED streaming and workflow pressures.

These findings suggest that reattendance patterns reflect the interplay of patient characteristics, presenting problems and local organisational processes rather than pathway allocation alone. They reinforce that SDEC provides a safe and effective route for suitable ambulatory presentations, with outcomes influenced by context-specific factors such as clinical judgement, operational routines, resource availability and patient selection.

Mortality was markedly lower in SDEC than in short-stay admissions, a difference that almost certainly reflects selection processes rather than a direct protective effect of the pathway. In the adjusted interaction model, patients managed via SDEC had substantially lower odds of 30-day mortality, consistent with the pathway being used for individuals with lower clinical acuity, despite the use of the comparator of short-stay patients as a proxy for low-acuity. Age and sex remained important predictors, with younger patients and women experiencing lower mortality risk. The interaction between pathway and sex indicated that the relative difference in mortality between SDEC and short-stay admission varied by sex, suggesting that clinical assessment and selection processes may operate differently for men and women. These findings support the interpretation that the observed mortality differences are driven primarily by underlying factors such as patient selection and clinical judgement rather than by intrinsic effects of the pathways. The results are consistent with SDEC operating as a safe model of care. Differences between the two hospitals further highlight the influence of local operational context on pathway outcomes.

Across all outcomes, the patterns observed are more likely to reflect differences in patient mix, triage decisions, organisational processes and wider system pressures than inherent differences between the pathways themselves. Because SDEC relies on initial ‘front-door’ identification of suitable ambulatory presentations, variation in triage models, whether based on clinical judgement, structured criteria^23^ or local operational constraints, plays a key role in determining who enters the pathway and how it performs. These findings highlight that pathway outcomes are shaped by both patient characteristics and the organisational context in which care is delivered, rather than inherent differences in the pathways. This is further supported by the fact that both hospitals followed the same SOP yet produced different patterns of pathway use, indicating that operational pressures and local workflow shape real-world implementation.

Published evidence on SDEC outcomes is limited,^4^ with only a small number of studies reporting non-comparable outcomes because of differences in setting, population and the period in which they were conducted. Reschen *et al*^7^ reported a higher mean conversion-to-admission rate, particularly among older adults, but their work predated the national SDEC strategy and was undertaken in an assessment unit rather than a dedicated SDEC ward, where admission to a bed may have been more straightforward. They also reported lower 30-day reattendance and mortality among ambulatory patients, with higher reattendance in those over 75 years, although results were presented graphically and their admitted cohort was not restricted by length of stay. Elias *et al*^8^ examined outcomes in an emergency assessment unit where an ambulatory pathway was available, but their cohort comprised older, frailer patients with increased care needs, falls, therapy requirements, severe illness or delirium, making their immediate admissions, 30-day readmissions and mortality rates not comparable to the present study. The national Society for Acute Medicine benchmarking audit reports only 7-day reattendance and mortality,^5^ and Jones *et al*^6^ found no evidence specifically addressing conversion, 30-day reattendance or mortality in acute medical SDEC populations. The lack of published evidence emphasises the relevance of the current study, which evaluates conversion, 30-day reattendance and mortality within a contemporary, dedicated SDEC model and a clearly defined short-stay cohort aligned with current national policy. To support evaluation of SDEC in the future, safety and quality metrics have been proposed.^25^ Although Jones *et al*^6^ highlighted the limited international evidence base for SDEC and related ambulatory emergency care models, emerging work from European systems provides useful context. Several countries, including Denmark, the Netherlands and Spain, operate comparable same-day or short-stay emergency care pathways, and these services report broadly similar patterns of low conversion to admission and low short-term mortality among ambulatory patients. As in our study, outcomes in these systems appear to be shaped more by local operational pressures, triage behaviour and workflow routines than by formal pathway criteria. This alignment suggests that the mechanisms identified here are not unique to the UK setting but reflect wider system-level dynamics observed internationally.

Overall, the reported study indicates that SDEC functions as a safe pathway, characterised by low conversion to admission and substantially lower 30-day mortality than short-stay admission, differences that appear to reflect patient selection rather than intrinsic protective effects of the pathway itself. Prefix-concordant reattendance was also lower among SDEC patients in the adjusted interaction model, suggesting that same-day discharge does not increase the likelihood of clinically related return visits and is consistent with safe management of ambulatory presentations. Variation in the SDEC effect by age and site highlights that outcomes are shaped by interactions between patient characteristics, clinical judgement and local organisational context. These patterns reinforce that pathway outcomes are emergent properties of the wider UEC system, shaped by workflow pressures, resource constraints and local service configuration, rather than fixed attributes of SDEC or short-stay admission. The results suggest practical implications for service design: pathway performance depends on how patients are selected, how teams are resourced and how local processes operate, suggesting that refinements to triage, staffing and diagnostic access may support more consistent and effective use of SDEC. The fact that both hospitals demonstrated different patterns of pathway use illustrates that implementation, not policy, drives performance.

This study has several methodological strengths. The use of a large, well-defined cohort drawn from routinely collected data allowed examination of SDEC outcomes across a 4-year period, capturing real-world practice within a pressured UEC system. Consistent inclusion criteria were applied, with rigorous data management, and sensitivity analyses enhanced internal validity and supported the robustness of the findings. The multivariable modelling strategy enabled exploration of interactions between patient characteristics, organisational context and pathway allocation.

Important limitations must be acknowledged. As with all analyses based on administrative data, coding accuracy and variable availability constrained the granularity of measurement. The administrative dataset records sex but does not include a gender variable; analyses were therefore limited to the available sex categories. Collapsing all non-White British groups into ‘other’ may obscure heterogeneity but it was necessary due to sparse counts. Socioeconomic variables were not available due to the nature of the dataset. Clinical observations and frailty scores at presentation were not available for analysis because, although they are recorded in paper notes, they are not captured in extractable electronic form. As manual review of all records was not feasible, short-stay admission was used as a pragmatic proxy for lower acuity. Case identification was incomplete because many acute medical patients managed entirely within the ED for up to 48 hours were not coded to the acute medical admission pathway. These patients are clinically equivalent to short-stay admissions but are not captured in the inpatient dataset, leading to under-representation of the short-stay cohort and making the proportion of patients managed through SDEC appear higher than it truly is. Outcome capture was also limited by the inability to identify mortality or reattendance events occurring outside the trust, although the geographical configuration of the county suggests that such events are likely to be few. Although the study was conducted in two hospitals within a single trust, the mechanisms identified, particularly the influence of patient selection, operational pressures and workflow routines, are common across NHS UEC systems and comparable international models. The findings are therefore conceptually generalisable, even if absolute rates may vary between settings.

Finally, the study reflects the configuration of SDEC and acute medical services across two sites within a single trust. During the study period, SDEC operated for 12–14 hours per day, meaning that patients presenting overnight were initially managed in the ED; although many were transferred to SDEC at 8am, this does not eliminate structural differences between pathways. Comparisons with ≤48-hour short-stay admissions may therefore reflect differences in service availability as well as clinical need. While organisational structures and referral thresholds vary across the NHS, the mechanisms identified, particularly those relating to patient selection, operational incentives and system-level pressures, are not unique to this setting. Similar dynamics shape UEC pathways across the NHS and in international systems implementing same-day or ambulatory emergency care models. The insights generated here are likely to have broader relevance for services seeking to optimise patient flow, manage demand, and provide safe alternatives to hospital admission.

These findings have implications for both national policy and local service design. The divergence between ED flow pressures and national SDEC specifications suggests that current policy expectations may not align with operational realities, particularly where SDEC is increasingly used to manage lower-acuity ED patients who are unlikely to require admission. At a national level, clarifying the intended function of SDEC within the UEC system, as has now been done but has yet to be embedded,^18 19^ and aligning performance metrics with real-world case-mix,^25^ may support more coherent pathway design. At the level of individual hospitals, our findings indicate that pathway performance is shaped by how national guidance is operationalised locally, particularly in relation to patient selection, ED streaming and workflow pressures. Further research should examine outcomes in relation to patient acuity at admission, referral decision-making, triage processes, and the impact of local service configurations, to better understand how SDEC is being used across different organisational contexts. Comparative studies across multiple trusts would help establish whether the outcomes observed for SDEC represent local effects or wider system-level patterns in patient care. Complementary qualitative work examining patient experiences of SDEC, reported separately, may help contextualise these patterns and clarify how patients perceive the purpose and value of SDEC within the wider UEC system.

## Conclusions

Overall, these findings indicate that SDEC functions safely and effectively within the UEC system for appropriately selected patients. Conversion to admission was low, suggesting that most patients managed through SDEC were safely discharged without requiring subsequent inpatient care. Prefix-concordant reattendance was also lower among SDEC patients, indicating that same-day discharge did not increase the likelihood of clinically related return visits. 30-day mortality was substantially lower in SDEC than in short-stay admissions, a pattern consistent with the selection of lower-acuity patients rather than a direct protective effect of the pathway. These results support SDEC as a safe model of care for ambulatory presentations within acute medicine.

## Supplementary material

10.1136/bmjopen-2026-121412 online supplemental file 1

## Data Availability

Data are available in a public, open access repository.
