# Supplementary material for: Clinical outcomes of adults accessing acute medical same-day emergency care in the NHS: a retrospective cohort study across two hospitals
Source: BMJ Open. 2026 Jul 1;16(7):e121412. doi: 10.1136/bmjopen-2026-121412 (PMC13331122; doi:10.1136/bmjopen-2026-121412)
Supplement: online supplemental file 1 [file bmjopen-16-7-s001.docx]

Supplemental material

The top 20 reasons for admission showed clear differences in diagnostic mix between pathways. Respiratory, cardiac and infection‑related presentations predominated overall, but their relative frequencies varied between SDEC and short‑stay admissions. Several lower‑volume diagnoses contributed small but heterogeneous proportions of activity. These patterns illustrate the diversity of case‑mix across pathways and the influence of presenting condition on subsequent patient flow.

*Supplementary Figure 1: Monthly Attendance Trend by Diagnosis and Care Pathway*


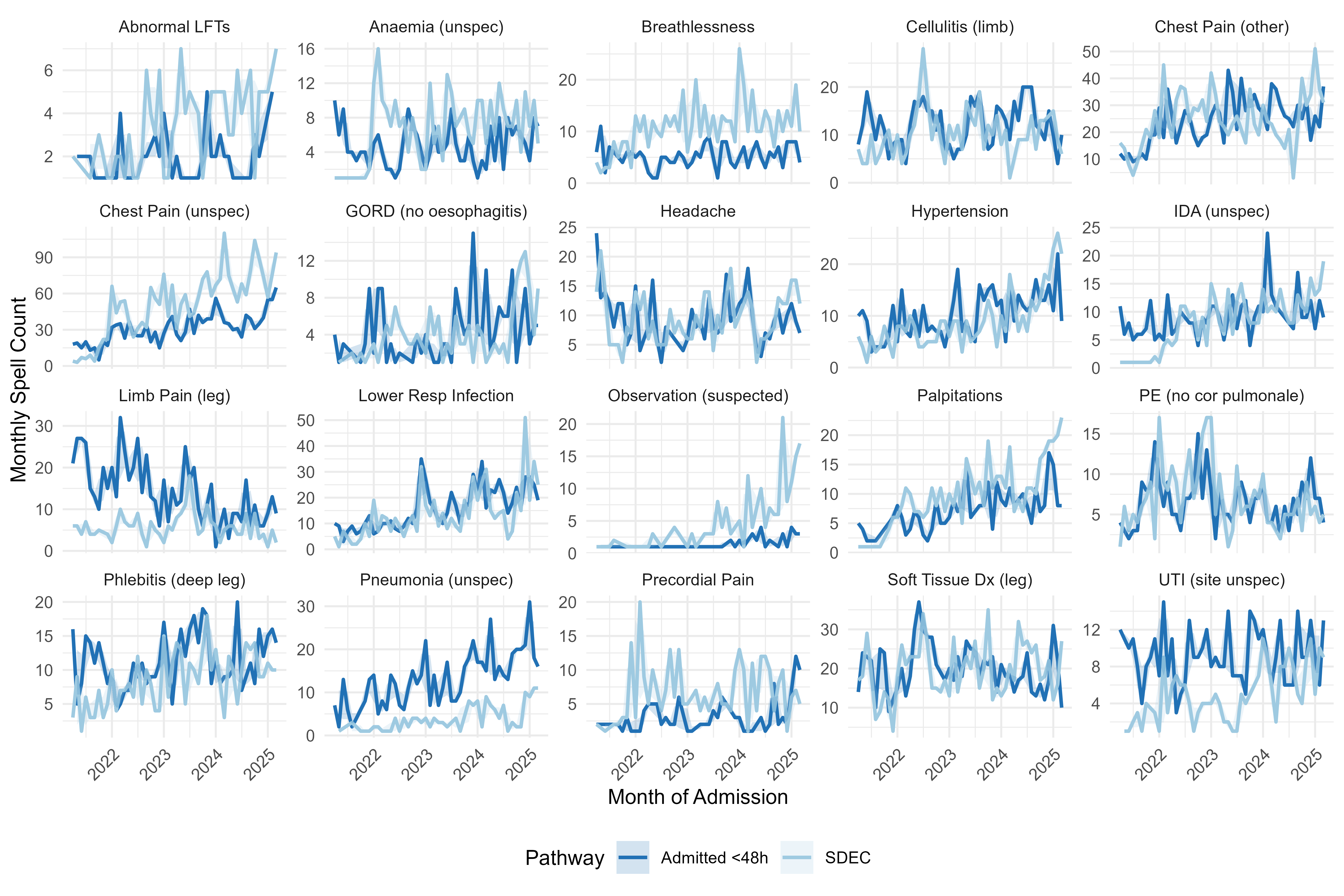


Note: Monthly attendance counts are based on first admissions per patient per calendar month. Repeat admissions within the same month are excluded. Pathway classification reflects the most recent coding available at time of admission. Reclassifications post-discharge are not applied. confidence intervals are shown as shaded ribbons. Intervals are suppressed where monthly n < 30 to avoid unreliable estimates. Facet scales are independent across diagnoses to preserve within-group variation. Y-axis comparisons across panels are not recommended. Data exclude attendances with missing or ambiguous diagnosis codes, pathway assignments, or admission dates. Trends may reflect both true variation and changes in coding, service configuration, or data completeness over time.
