# Supplementary material for: Clinical outcomes of adults accessing acute medical same-day emergency care in the NHS: a retrospective cohort study across two hospitals
Source: BMJ Open. 2026 Jul 1;16(7):e121412. doi: 10.1136/bmjopen-2026-121412 (PMC13331122; doi:10.1136/bmjopen-2026-121412)
Supplement: Supplementary file 2 [file bmjopen-2026-121412.reviewer_comments.pdf]

PEER REVIEW HISTORY

BMJ Open publishes all reviews undertaken for accepted manuscripts. Reviewers are asked to complete a checklist review form and are provided with free text boxes to elaborate on their assessment. These free text comments are reproduced below.

ARTICLE DETAILS

Title (Provisional)

Clinical Outcomes of Adults Accessing Acute Medical Same Day Emergency Care in the NHS: A Retrospective Cohort Study Across Two Hospitals

Authors

Dean, Sue; Leonardi-Bee, Jo; Barratt, Julian; Blake, Holly

VERSION 1 - REVIEW

**Reviewer**	**1**
**Name**	**Subbe, Christian**
**Affiliation**	**Bangor University, North Wales Medical School**
**Date**	**16-May-2026**
**COI**	**None**

Many thanks for the opportunity to review this paper from two general hospitals in the UK that compares outcomes of patients from two pathways: patients seen in Same Day Emergency Care Units (SDEC) and low risk admissions defined as patients discharged within 48 hours.

Patient seen in SDEC had lower mortality, lower re-admission rates and low admission rates.

It appears that the proportion of patients going through SDEC is high - nationally about a third of all referrals to the department of medicine will go through SDECs, in the study units it appears to be more than two thirds but I have not seen enough of the referral patterns from primary care or those presenting directly to the Emergency Department.

While the study documents safe functioning of the pathways in the two study hospitals I am unclear about what the learning for other hospitals would be?

I might have missed information about the make-up of the hospitals, service set up and referral criteria.

It is unclear to me how well the two cohorts were matched? For the Society for Acute Medicine's Benchmarking Audit (SAMBA) data is stratified by severity of illness as measured by the National Early Warning Score and level of frailty as measured by the Clinical Frailty Scale. Co-morbidity (indexed or otherwise) and mobility are massive factor for 'ambulatory'

patients in SDEC so again without this data it is difficult to interpret the findings of the authors and for others to learn from the experience of the team.

If would be fantastic to have a system where 95% of patients go home and this makes up two thirds of the take straight from referral. But this data is not contained in the paper. This type of analysis would be useful but require significantly more in depth statistical analysis.

Reviewer 2

Name Zakariassen, Erik

Affiliation Universitetet i Bergen Det medisinsk-odontologiske fakultet, Department of Global Public Health and Primary Care

Date 23-May-2026

COI None

Clinical Outcomes of Adults Accessing Acute Medical Same Day Emergency Care in the NHS: A Retrospective Observational Cohort Study Across Two Hospitals.

Overall, this is an interesting study. Different countries have different ways of dealing with patients with low severity, but they still attend hospital EDs. The study describes one way to address the low-severity patient group.

When I refer to page numbers, I am referring to the page numbering displayed at the top of each page (e.g., “Page 5 of 23”).

Introduction

There is a need for a better description of the selection method of patients in the two hospitals. This should be written in the introduction or in the study setting. For readers outside the UK, it is important to have this explained early. Do all patients meet at the ED in Boston and Lincoln, and after some kind of triage, are moved to the Same Day Emergency Care (SDEC)?

The paragraph that starts on line 12 (page 5) should be moved to the study section.

Line 15, (see ‘Ethics’ approval’ section for the justification of citing the names of the research sites) is not needed.

Line 26, page 5. The sentence, “This paper reports the quantitative component of a wider doctoral fellowship programme,” is not needed.

Method

Line 39, page 5. “Only the first SDEC attendance was included, with follow-up visits being excluded”. This is some confusing. Reattendance is measured, but follow-up visits are not reattendance? Please clarify what follow-up visits are. I find the same problem with the

sentence in line 56, “Repeat attendances after the index visit, and SDEC visits undertaken solely for post-discharge review, were not provided in the dataset, as they were excluded at source”. I understand “…SDEC visits undertaken solely for post-discharge review”, but “repeated attendance after the index visit” is that not reattendance? Please clarify this.

Results

There is no need to present figures both in text and in tables. Please reduce the presentation of figures in the text when we can see the figures in the tables. The same for presentation of OD and 95% CI. If I find this in a table, I do not need it in the text. This will shorten the text and make the presentation easier to read. In the text, highlight what's important in connection with the different tables.

Discussion

Overall, the discussion is thorough. Still, I miss a more international comparison/discussion of the results. Are there no studies on other European systems that can be useful to discuss against the results?

Line 8, page 13 – A structural conflict between the recommendation and the pressure to reduce crowding. Is this really a conflict? In my context, you can follow a recommendation, but it is not mandatory. This contrasts with a regulation or law that you must follow. Is there any consequence if the recommendations are not followed?

Line 17, page 13 – Patient selection for SDEC. As mentioned earlier, how are the patients selected at Boston and Lincoln Hospitals? You discuss the results on a general basis. Please also discuss the results in relation to how the patient selection was performed in the two hospitals. This is important due to the results showing large differences between the two hospitals in SDEC and admitted patients.

Line 6, page 14 - hole paragraph. Is this a general discussion of the UK system or a discussion of the two hospitals? Please clarify.

Conclusion

My suggestion is to start with sentence 15, “Overall, these findings indicate that SDEC functions safely and effectively within the UEC system for appropriately selected patients”. The rest of the paragraph, starting with line 15, is a discussion, not a conclusion.

**Reviewer**	**3**
**Name**	**Gilbert, Allison**
**Affiliation**	**University Hospital Center Liege, Emergency Medicine**
**Date**	**27-May-2026**
**COI**	**None**

Overall comment:

This retrospective study explored the "Same Day Emergency Care" (SDEC) model in terms of 30-day patient reattendance and 30-day mortality, compared with the conventional pathway of care. This is an interesting topic, but several concepts need to be refined to improve the overall clarity of the manuscript. Also, the structure of the article should be improved. Considering this, I would like to raise a number of major and minor comments.

Major comments:

Introduction section:

The concept of SDEC should be more clearly defined to improve the clarity of the manuscript. Several elements of the introduction relate more to methodological aspects or to the study's context and should therefore be moved to the methods section (see p4, lines 25–26; p4, lines 12–15). In addition, reports of other SDEC models could be described in the introduction to provide a broader perspective on the concept.

Methods section:

A more detailed explanation of how the two-site SDEC is organised is needed for clarity. A process flow diagram could also strengthen the manuscript. Have the authors considered accounting for clinical severity using a validated scoring system?

Results section:

Some of the figures appear to diverge (full cohort); could the authors please verify these numbers?

Minor comments:

How generalisable are these findings? As this is a two-site study, the results may not be applicable to all settings. Please consider this through the whole manuscript.

VERSION 1 - AUTHOR RESPONSE

Reviewer: 1
Dr. Christian Subbe, Bangor University
Comments to the Author:

Many thanks for the opportunity to review this paper from two general hospitals in the UK that compares outcomes of patients from two pathways: patients seen in Same Day Emergency Care Units (SDEC) and low risk admissions defined as patients discharged within 48 hours. Patient seen in SDEC had lower mortality, lower re-admission rates and low admission rates. It appears that the proportion of patients going through SDEC is high - nationally about a third of all referrals to the department of medicine will go through SDECs, in the study units it appears to be more than two thirds but I have not seen enough of the referral patterns from primary care or those presenting directly to the Emergency Department.

Response: Thank you for raising this point. We agree that the proportion of patients managed through SDEC appears high relative to national estimates. One contributing factor is the challenge of accurately identifying all acute medical short-stay patients within our Trust. A substantial number of patients remained in the ED under specialty for up to 48 hours but are not coded as inpatients due to local coding processes. These patients are clinically equivalent to short-stay admissions but are not captured in the inpatient dataset, which reduces the apparent size of the short-stay cohort and inflates the relative proportion of SDEC activity. Our study therefore reflects the coded short-stay population rather than the full denominator of acute medical patients managed within 48 hours. This is clarified in the limitations paragraph. We did not specifically consider referral pathways.

While the study documents safe functioning of the pathways in the two study hospitals I am unclear about what the learning for other hospitals would be?

Response: Thank you for this helpful comment. We have strengthened the Discussion to clarify the implications for other hospitals. We now explicitly describe how the mechanisms identified, particularly patient selection, operational pressures, and local workflow structures, are relevant to other NHS trusts and to international systems implementing ambulatory emergency care. We emphasise that the findings highlight *how* SDEC functions safely, and under what organisational conditions, rather than being specific to the two study sites.

I might have missed information about the make-up of the hospitals, service set up and referral criteria.

Response: We have expanded the Study Setting section to describe the two hospital sites, referral pathways, and the operational criteria used to select patients for SDEC.

It is unclear to me how well the two cohorts were matched? For the Society for Acute Medicine's Benchmarking Audit (SAMBA) data is stratified by severity of illness as measured by the National Early Warning Score and level of frailty as measured by the Clinical Frailty Scale. Co-morbidity (indexed or otherwise) and mobility are massive factor for 'ambulatory' patients in SDEC so again without this data it is difficult to

interpret the findings of the authors and for others to learn from the experience of the team.

Response: We have expanded the description of the services and pathways and agree that clinical severity and frailty are important factors. These measures were not available for analysis because, although NEWS2 and frailty scores are recorded in paper notes, they are not captured in extractable electronic form within the Trust. Manual review of all records was not feasible for this large cohort. We have now added this clarification to the Study Design and Setting section and expanded the Limitations section. Propensity score matching was considered but was not feasible within the resources of a single-researcher PhD project.

If would be fantastic to have a system where 95% of patients go home and this makes up two thirds of the take straight from referral. But this data is not contained in the paper. This type of analysis would be useful but require significantly more in depth statistical analysis.

Response: We agree that understanding the proportion of the total acute medical take managed through SDEC, and the proportion discharged the same day, would provide valuable system-level insight. However, the present study was not designed to evaluate the full acute medical take or referral patterns into SDEC.

As we clarify in the Discussion, our SDECs currently receive a substantial number of patients streamed from the ED who do not require acute medical specialty input. These patients are referred primarily to relieve ED crowding rather than because they meet SDEC criteria. As a result, many are low-acuity and are discharged the same day, which increases the overall SDEC discharge rate and broadens the case-mix beyond that expected in a criteria-based ambulatory model.

A whole-system evaluation of referral pathways, case mix and admission-avoidance potential would require a broader dataset and more complex modelling. Work of this kind is being undertaken by research teams at the University of Sheffield, who are leading national analyses of urgent and emergency care pathways.

Reviewer: 2
Dr. Erik Zakariassen, Universitetet i Bergen Det medisinsk-odontologiske fakultet, Uni Research AS
Comments to the Author:
Clinical Outcomes of Adults Accessing Acute Medical Same Day Emergency Care in the NHS: A Retrospective Observational Cohort Study Across Two Hospitals.
Overall, this is an interesting study. Different countries have different ways of dealing with patients with low severity, but they still attend hospital EDs. The study describes one way to address the low-severity patient group.
When I refer to page numbers, I am referring to the page numbering displayed at the top of each page (e.g., "Page 5 of 23").
Introduction

There is a need for a better description of the selection method of patients in the two hospitals. This should be written in the introduction or in the study setting. For readers outside the UK, it is important to have this explained early. Do all patients meet at the ED in Boston and Lincoln, and after some kind of triage, are moved to the Same Day Emergency Care (SDEC)?

Response: We have added a clear description of the patient referral and selection criteria used locally for SDEC. This information is now included in the Study Setting section.

The paragraph that starts on line 12 (page 5) should be moved to the study section.
Response: This paragraph has been removed from the Introduction and relocated to the Study Setting section as requested.

Line 15, (see 'Ethics' approval' section for the justification of citing the names of the research sites) is not needed.
Response: This sentence has been removed.

Line 26, page 5. The sentence, "This paper reports the quantitative component of a wider doctoral fellowship programme," is not needed.
Response: This sentence has been removed.

Method
Line 39, page 5. "Only the first SDEC attendance was included, with follow-up visits being excluded". This is some confusing. Reattendance is measured, but follow-up visits are not reattendance? Please clarify what follow-up visits are. I find the same problem with the sentence in line 56, "Repeat attendances after the index visit, and SDEC visits undertaken solely for post-discharge review, were not provided in the dataset, as they were excluded at source". I understand "...SDEC visits undertaken solely for post-discharge review", but "repeated attendance after the index visit" is that not reattendance? Please clarify this.

Response: We have now clarified this distinction in the Study Design and Setting section. Planned follow-up reviews arranged by SDEC clinicians were excluded at source, while unplanned reattendance within 30 days was captured as an outcome.

Results
There is no need to present figures both in text and in tables. Please reduce the presentation of figures in the text when we can see the figures in the tables. The same for presentation of OD and 95% CI. If I find this in a table, I do not need it in the text. This will shorten the text and make the presentation easier to read. In the text, highlight what's important in connection with the different tables.

Response: Thank you for this helpful suggestion. We have revised the Results section extensively to remove duplicated numerical values where these are already presented in the tables. The text now focuses on the key patterns and differences between groups,

with detailed figures retained in the tables. We believe this has substantially improved clarity and readability. All changes have been highlighted in the revised manuscript.

Discussion
Overall, the discussion is thorough. Still, I miss a more international comparison/discussion of the results. Are there no studies on other European systems that can be useful to discuss against the results?

Response: We agree that situating the findings within an international context strengthens the Discussion. We have now included an international comparison paragraph. This addition situates our findings within a wider international context.

Line 8, page 13 – A structural conflict between the recommendation and the pressure to reduce crowding. Is this really a conflict? In my context, you can follow a recommendation, but it is not mandatory. This contrasts with a regulation or law that you must follow. Is there any consequence if the recommendations are not followed?

Response: Thank you for raising this point. We agree that national SDEC recommendations are not mandatory, and therefore "conflict" is too strong. Our intention was to describe the operational tension between ED crowding pressures and national expectations for SDEC case-mix. We have replaced "structural conflict" with "practical tension" to reflect this more accurately.

Line 17, page 13 – Patient selection for SDEC. As mentioned earlier, how are the patients selected at Boston and Lincoln Hospitals?

Response: We have expanded the Study Setting section to describe referral routes (ED triage, ambulance and GP referral) and local eligibility criteria. This section also now clarifies that clinical observations and frailty scores were not available electronically and therefore could not be included in the analysis.

You discuss the results on a general basis. Please also discuss the results in relation to how the patient selection was performed in the two hospitals. This is important due to the results showing large differences between the two hospitals in SDEC and admitted patients.

Response: Thank you for this helpful point. Both hospitals operated under the same Standard Operating Procedure for SDEC throughout the study period. We have clarified this in the Discussion. Despite identical formal criteria, the two sites differed in operational pressures, ED streaming practices and local workflow routines, which likely contributed to the observed variation in conversion rates, reattendance and mortality. We have expanded the Discussion to reflect how these site-level contextual factors shaped patient selection in practice.

Line 6, page 14 - hole paragraph. Is this a general discussion of the UK system or a discussion of the two hospitals? Please clarify.

Response: Thank you for highlighting this. We've rewritten the paragraph, so it is clear which parts refer to the wider UK system, and which relate specifically to the two study hospitals. The revised version now separates national policy context from local operational factors, making the scope of the discussion much clearer.

Conclusion
My suggestion is to start with sentence 15, "Overall, these findings indicate that SDEC functions safely and effectively within the UEC system for appropriately selected patients". The rest of the paragraph, starting with line 15, is a discussion, not a conclusion.

Response: Thank you for this helpful suggestion. We have revised the Conclusion so that it now opens with the sentence you highlight and focuses solely on summarising the key findings. The material that was more interpretive has been removed to avoid overlap.

Reviewer: 3
Dr. Allison Gilbert, University Hospital Center Liege
Comments to the Author:
Overall comment:
This retrospective study explored the "Same Day Emergency Care" (SDEC) model in terms of 30-day patient reattendance and 30-day mortality, compared with the conventional pathway of care. This is an interesting topic, but several concepts need to be refined to improve the overall clarity of the manuscript. Also, the structure of the article should be improved. Considering this, I would like to raise a number of major and minor comments.

Major comments:
Introduction section:
The concept of SDEC should be more clearly defined to improve the clarity of the manuscript. Several elements of the introduction relate more to methodological aspects or to the study's context and should therefore be moved to the methods section (see p4, lines 25–26; p4, lines 12–15). In addition, reports of other SDEC models could be described in the introduction to provide a broader perspective on the concept.

Response: We have strengthened the opening paragraph of the Introduction by adding a clearer definition of SDEC and its purpose. We have added a brief sentence referencing comparable international models to broaden the context. All methodological and site-specific content has been removed from the Introduction and moved.

Methods section:
A more detailed explanation of how the two-site SDEC is organised is needed for clarity.

Response: The Study Design and Setting section has been expanded to explain how SDEC operates.

A process flow diagram could also strengthen the manuscript.

Response: Thank you for this suggestion. We agree that a process flow diagram can be a useful way to illustrate pathway structure. In this case, we have aimed to describe the referral routes, SDEC processes and short-stay pathways clearly within the text, and we feel that the narrative already provides sufficient detail for readers to understand the flow of patients through the system. We therefore have not added a diagram at this stage but would be happy to consider this in future work where pathway redesign or implementation processes are the primary focus.

Have the authors considered accounting for clinical severity using a validated scoring system?

Response: We agree that severity scoring would strengthen the analysis. NEWS2 and frailty scores are recorded in paper notes within the Trust but are not captured electronically, making extraction at scale infeasible. We have added this explanation to the Study Design and Setting section and expanded the Limitations to reflect this constraint and its implications for interpretation.

Results section:
Some of the figures appear to diverge (full cohort); could the authors please verify these numbers?

Response: Thank you for highlighting this. We have re-checked all figures and can confirm that the numbers are correct. The apparent divergence reflects the use of different denominators for different analyses. The short-stay analytic cohort (n=43,970) includes patients initially managed in SDEC or admitted for ≤48 hours. In contrast, the conversion-to-admission analysis uses the full SDEC attendance cohort (n=29,186), which includes all patients who arrived in SDEC, including those subsequently admitted, regardless of short-stay eligibility. Reattendance and mortality analyses are restricted to the short-stay analytic cohort. We have clarified these distinctions in the Results section.

Minor comments:
How generalisable are these findings? As this is a two-site study, the results may not be applicable to all settings. Please consider this through the whole manuscript.

Response: We have strengthened the Discussion to clarify that although both hospitals used the same SOP, differences in operational context produced variation in pathway performance. This reinforces that the mechanisms identified, particularly patient selection, workflow pressures and ED streaming, are likely to operate across other NHS settings, supporting conceptual generalisability even if absolute rates vary.

VERSION 2 - REVIEW

**Reviewer**	**2**
**Name**	**Zakariassen, Erik**
**Affiliation**	**Universitetet i Bergen Det medisinsk-odontologiske fakultet, Department of Global Public Health and Primary Care**
**Date**	**04-Jun-2026**
**COI**	

The manuscript is better. I have just one comment regarding repetition of text. In the discussion, there is a repetition of information; both hospitals operated under the same SOP … "both hospitals used the same SOP… "despite identical SOPs…

Also, in the results section, there is a repetition of the method; multivariable logistic regression was used to examine adjusted associations.

The authors should read through the manuscript and make sure that repetition of information is reduced.

**Reviewer**	**3**
**Name**	**Gilbert, Allison**
**Affiliation**	**University Hospital Center Liege, Emergency Medicine**
**Date**	**15-Jun-2026**
**COI**	

The authors answered adequately to all my previous comments.

VERSION 2 - AUTHOR RESPONSE

Response to Reviewer Comments:

Thank you for the opportunity to revise the article and for the helpful comments.

Reviewer 2:

In the discussion, there is a repetition of information; both hospitals operated under the same SOP ... "both hospitals used the same SOP... "despite identical SOPs...
Also, in the results section, there is a repetition of the method; multivariable logistic regression was used to examine adjusted associations.

The authors should read through the manuscript and make sure that repetition of information is reduced.

Thank you, we have addressed the repetition.
